# Efficacy of prolotherapy in comparison to other therapies for chronic soft tissue injuries: A systematic review and network meta-analysis

**DOI:** 10.1371/journal.pone.0252204

**Published:** 2021-05-26

**Authors:** Siew-Li Goh, Zulkarnain Jaafar, Yan-Nee Gan, Alston Choong, Jaspreet Kaur, Burak Kundakci, Samihah Abdul Karim, Muhammad Rahmani Jaffar, Mohamad Shariff A. Hamid

**Affiliations:** 1 Sports and Exercise Medicine Research and Education Group, University of Malaya, Kuala Lumpur, Malaysia; 2 Malaysian Health Technology Assessment Section (MaHTAS), Ministry of Health, Kuala Lumpur, Malaysia; 3 Arthritis Research UK Pain Centre, University of Nottingham, Nottingham, United Kingdom; Universidad de Antioquia, COLOMBIA

## Abstract

**Introduction:**

Prolotherapy and other injections, primarily acting on pathways associated with maladaptive tissue repair, are recommended for recalcitrant chronic soft tissue injuries (CSTI). However, selection of injection is challenging due to mixed results. This network meta-analysis (NMA) aimed to compare prolotherapy with other therapies, particularly injections, for CSTI and establish robustness of the results.

**Methodology:**

Pubmed, Medline, SPORTDiscus and Google scholar were searched from inception to 4^th^ January 2021 for randomised controlled trials (RCTs) involving injection therapies (e.g. blood derivatives, corticosteroid, hyaluronic acid, botulinum toxin) for CSTI. The primary and secondary outcomes were pain and function, respectively, at (or nearest to) 6 months. Effect size (ES) was presented as standardised mean difference with 95% confidence interval (CI). Frequentist random effect NMA was used to generate the overall estimates, subgroup estimates (by region and measurement time point) and sensitivity analyses.

**Results:**

A total of 91 articles (87 RCTs; 5859 participants) involving upper limb (74%), lower limb (23%) and truncal/hip (3%) injuries were included. At all time points, prolotherapy had no statistically significant pain benefits over other therapies. This observation remained unchanged when tested under various assumptions and with exclusion of studies with high risk of bias. Although prolotherapy did not offer statistically significant functional improvement compared to most therapies, its ES was consistently better than non-injections and corticosteroid injection for both outcomes. At selected time points and for selected injuries, prolotherapy demonstrated potentially better pain improvement over placebo (<4 months: shoulder [ES 0.65; 95% CI 0.00 to 1.30]; 4–8 months: elbow [ES 0.91; 95% CI 0.12 to 1.70]; >8 months: shoulder [ES 2.08; 95% CI 1.49, to 2.68]). Injections generally produced greater ES when combined with non-injection therapy.

**Conclusion:**

While clinical outcomes were generally comparable across types of injection therapy, prolotherapy may be used preferentially for selected conditions at selected times.

## Introduction

Conditions attributed to overuse such as tendinopathy or enthesopathy qualify as chronic soft tissue injuries (CSTI), for which microtrauma from submaximal (but repetitive) loading had accrued over prolonged period. Such injuries are typically insidious without identifiable cause and often present only when physical activity is impaired by pain. Presentation is typically preceded by a long history of vague symptoms such as activity-related pain and stiffness. Homeostatic failure in tissue repair is cited as the primary mechanism driving the spectrum of clinical presentation and histopathological changes seen in the injured tissues [1, 2].

Since chronic injuries were being described as continuum of clinical pathologies more than 20 years ago [3], much advances had been achieved. Based on the dominant pathological feature, the continuum is now divided into three definitive phases: reactive, dysrepair and degenerative [4]. Transition from one phase to another would necessitate a corresponding change in pain management, whereby treatment strategy would shift from controlling inflammation in the early phase to promoting tissue healing in the later phase.

Despite the improved characterization of the continuum model in CSTI, it is still challenging to select the right treatment for few reasons. Firstly, clinical differentiation of the underlying pathology is difficult because symptoms do not always correlate with the degree of tissue damage [5]. Secondly, the different phases could overlap such as when reactive changes occur in the background of degenerated tendon [4]. Furthermore, the exact mechanism of action for some the treatments are still poorly understood. It is unclear where along the continuum does the therapeutic potential for these treatments lie. By and large, conservative therapy including injection is the mainstay of treatment for CSTI [6]. Non-invasive treatments (e.g. oral or local analgesics, exercises, taping and orthotics) and injection therapies (e.g. blood derivatives and prolotherapy) prescribed in CSTI aim to improve pain and function either directly, or indirectly by restoring normal intrinsic tissue morphology or correcting abnormal external loading [7].

Prolotherapy is one of the regenerative injection technique commonly used in various chronic musculoskeletal conditions [8]. The earliest evidence of its ‘proliferative’ effect was documented in the 1950’s for the treatment of ligament injuries [9]. Prolotherapy uses a small volume of sclerosant (0.5–6 ml) [10], such as hyperosmolar dextrose (>10%), polidocanol, glycerin, or phenol, to produce inflammatory reaction which then initiates the healing cascade. Due to its safety profile, cost-effectiveness and water-soluble property, hyperosmolar dextrose is the most common injectant selected for administering prolotherapy. Other regenerative injection therapies such as platelet rich plasma (PRP) and hyaluronic acid (HA) are also showing promise [11, 12]. However, there is currently no clear advantage between one treatment over another and comparisons are normally made in pairwise manner [13].

Considering that failed tissue healing underpins the pathogenesis of CSTI, there is a need to explore the efficacy of regenerative therapies in its management. The aim of this study was to compare the efficacy of prolotherapy with other therapies commonly used for CSTI, particularly focusing on injection therapies. We used network meta-analysis (NMA) to synthesize evidence on multiple treatments in CSTI. NMA is advantageous over conventional pairwise meta-analysis because it allows multiple treatments to be compared simultaneously, producing conclusion that is more cohesive.

## Methodology

The protocol for this study had been registered in PROSPERO (CRD42020149740). The conduct and reporting of this NMA was performed according to the PRISMA NMA checklist (S1 Table).

### Search strategy and selection criteria

Systematic search was performed using several databases: Pubmed, Medline, SPORTDiscus and Google scholar from inception to October 2019 with no language restrictions. The final update of the search was performed on 4th January 2021. An example of search strategy used is shown in S2 Table.

Eligibility criteria were as follows:

- The study was identified as randomised controlled trial (RCT);
- The soft tissue injuries were chronic (e.g., tendinopathies and enthesopathies) with mean symptom duration ≥ six weeks at study level;
- At least one intervention group received an injection therapy; and
- Pain or functional outcome were reported.

Studies were excluded if:

- The number of recruitments was less than 10 in at least two of the intervention groups;
- The injury was described as acute, irrespective of symptom duration;
- Injection therapy was given intra-articularly or into the bursa (based on description by the authors);
- There was no specific or identifiable tissue targeted for treatment (e.g., low back pain, shoulder impingement);
- The adjunct therapies between groups were not comparable (e.g. prolotherapy (Prolo) plus exercise vs. corticosteroids (CS) plus analgesics); or
- Outcome of interest was not extractable.

Screening was performed independently by at least two reviewers (SLG, ZJ, YNG) and all disagreements were resolved through consensus.

### Data extraction and assessment of study quality

The primary outcome of interest was pain. Functional outcome was used only for cluster analysis. Primary measurement time point was six months post-intervention. However, measurements at other times were also extracted to identify alternatives data point when six months outcome was not available. For studies with less than six months follow up, the longest time point was used. When more than one tool had been used to measure an outcome, data extraction was performed according to the hierarchy of measurement tool outlined in the protocol.

Conservative approaches were made whenever missing data or unclear reporting was encountered. For example, missing variance was imputed by identifying the widest value (e.g. standard deviation (SD)) from other studies with corresponding time points using the same measurement tool. Per-protocol analysis was assumed whenever the information provided was deemed insufficient to support intention-to-treat (ITT) analysis.

Other forms of data dispersion measurement such as range or interquartile range were converted to SD following method described by Wan et al [14]. When the number of participants included in the analysis was unclear, the largest number–normally corresponding to the number randomised–was used.

The quality of individual studies published in English was assessed using RoB 2.0 [15]. The certainty of evidence for direct comparisons between prolotherapy and other therapies was assessed using the Grading of Recommendations Assessment, Development and Evaluation (GRADE) approach [16]. The GRADE assessment was performed only for the primary outcome of pain and at the primary time point of 6 months. The summary and assessment of the certainty of findings using five domains (i.e. study design, risk of bias, inconsistency, indirectness and imprecision), were performed using the template from the GRADE’s software—GRADEpro GDT. Data screening and extraction was performed by SLG while other authors (YNG, WKC, JK, SAK, MRJ, MSAH) repeated similar process independently using a piloted and abridged form. Disagreements were resolved through consensus.

### Data synthesis and statistical analysis

#### Primary analysis

In the primary analysis, injections (e.g. CS, Prolo), were analysed based on their respective therapeutic properties. Blood derivatives (i.e. PRP, whole blood) were collectively analysed as blood products (BP). The suffix “combo” was used to denote combination of injections with other therapies (e.g. CScombo, BPcombo) (Table 1). Non-injection treatments (e.g. orthosis, physiotherapy, exercise) were aggregated as non-injections (Noninj). All forms of placebo and sham therapy (irrespective of delivery mode) were analysed as a single group (Pcb). Wait-see policy (Waiting) remained as a group on its own as it was in effect an observational group with no intervention. Both nodes and edges in the network geometry were weighted according to the number of studies involved in each treatment and comparison, respectively.

**Table 1:** Therapy grouping method for primary and secondary analyses.

Study design	Primary analysis	Secondary (1)	Secondary (2)
**Study 1 **			
**G1** Prolo + exercise	Prolocombo	Prolocombo	Combination
**G2** Prolo	vs. Prolo	vs. Prolo	vs. Inj
**G3 **Education	vs. Noninj	vs. Noninj	vs. Noninj
**Study 2 **			
**G1** Prolo + exercise	Prolo	Prolocombo	Inj
**G2** NSAIDs + exercise	vs Noninj	vs Noninjcombo	vs Noninj
**G3** Inert pill + exercise	vs Pcb	vs Pcbcombo	vs Pcb2
**Study 3 **			
**G1** Prolo	Prolo	Prolo	Excluded
**G2** Whole blood	vs. BP	vs. WB	
**G3** PRP		vs. PRP	

Note: G = study group; Inj = injection; noninj = Non-injection; NSAIDs = non-steroidal anti-inflammatory drugs; Prolo = prolotherapy; Pcb = placebo; Pcb2 = placebo Non-injection; PRP = platelet rich plasma; combo = combination therapy; WB = whole blood

Follow-up score was used to calculate standardised mean difference between groups as a measure of treatment effect size (ES). Change score within and between group was used when follow-up score was not available. The final analysis was performed using STATA 16.1 based on frequentist random effects model.

#### Secondary and sensitivity analysis

Sensitivity analysis was performed to assess the changes in summary estimates under different assumptions and moderators listed below:

- using change score as primary data;
- subgroup analysis by different time points (i.e. <4 months, 4 to <8 months and ≥8 months post intervention) for
  - overall (i.e. all soft tissue conditions) estimate,
  - by region: lateral/ medial epicondylitis and chronic rotator cuff injuries;
- excluding studies with high risk of bias.

Secondary analyses were performed to explore efficacy of prolotherapy under two grouping alternatives. The first secondary analysis (secondary 1) adopted a grouping approach aimed to distinguish efficacy between single and combination therapies and between different types of blood products. The second secondary analysis (secondary 2), on the other hand, adopted a grouping approach that aimed to compare the efficacy between injection and non-injection delivery methods. In secondary analysis 2, placebos were divided into injection and non-injection (i.e. Pcb1 –injection with non-active agents; Pcb2 –non-injection) to verify the observation of Bannuru et al [17] that injection therapies confer greater treatment effects than non-injectables. The different approaches in grouping for the primary and secondary analyses are summarised in Table 1.

*Primary analysis*. Primary analysis aimed to compare prolotherapy with other treatments as a collective group (i.e. blood products as a group, non-injections as a group, all placebos as a group). Therapies with adjuncts would be denoted with a suffix ‘combo’ (e.g. G1 Study 1), unless the adjunctive therapies had been standardised for all study groups (e.g. Study 2 where the exercise adjunct was omitted from primary analysis).

*Secondary (1)*. Secondary analysis aimed to assess the efficacy of single or combination therapy and to explore effects of different blood products. Therefore, all study groups given adjunctive therapies were analysed as ‘combo’ irrespective of whether the adjunctive treatments were standardised in the study (e.g. G1, G2 and G3 Study 2). Also, unlike primary analysis, types of blood products were analysed individually and were not aggregated (e.g. G2 and G3 Study 3). However, non-injections remained as an aggregated group (e.g. G3 Study 1, G2 Study2).

*Secondary (2)*. Secondary analysis aimed to assess the efficacy with injectables versus non-injectables. Therapies, and also placebos, given via injections were separated from those given non-invasively. When groups within the same study all used the same delivery mode (e.g. Study 3), the study would not be eligible for inclusion in this analysis.

### Cluster analysis

Using measurement time points between 4–8 months, an additional cluster analysis was undertaken to explore the efficacy of all treatments for the joint outcomes of pain and function improvement. The purpose was to assess the treatment effect on pain and functional outcome simultaneously.

### Model checking

We used node-splitting method to assess consistency between direct and indirect evidence. Study design inconsistency was assessed using network forest plot by design. The primary analysis was repeated post-hoc by fitting the inconsistency model. Heterogeneity was assessed based on between studies SD.

## Results

**Fig 1:**
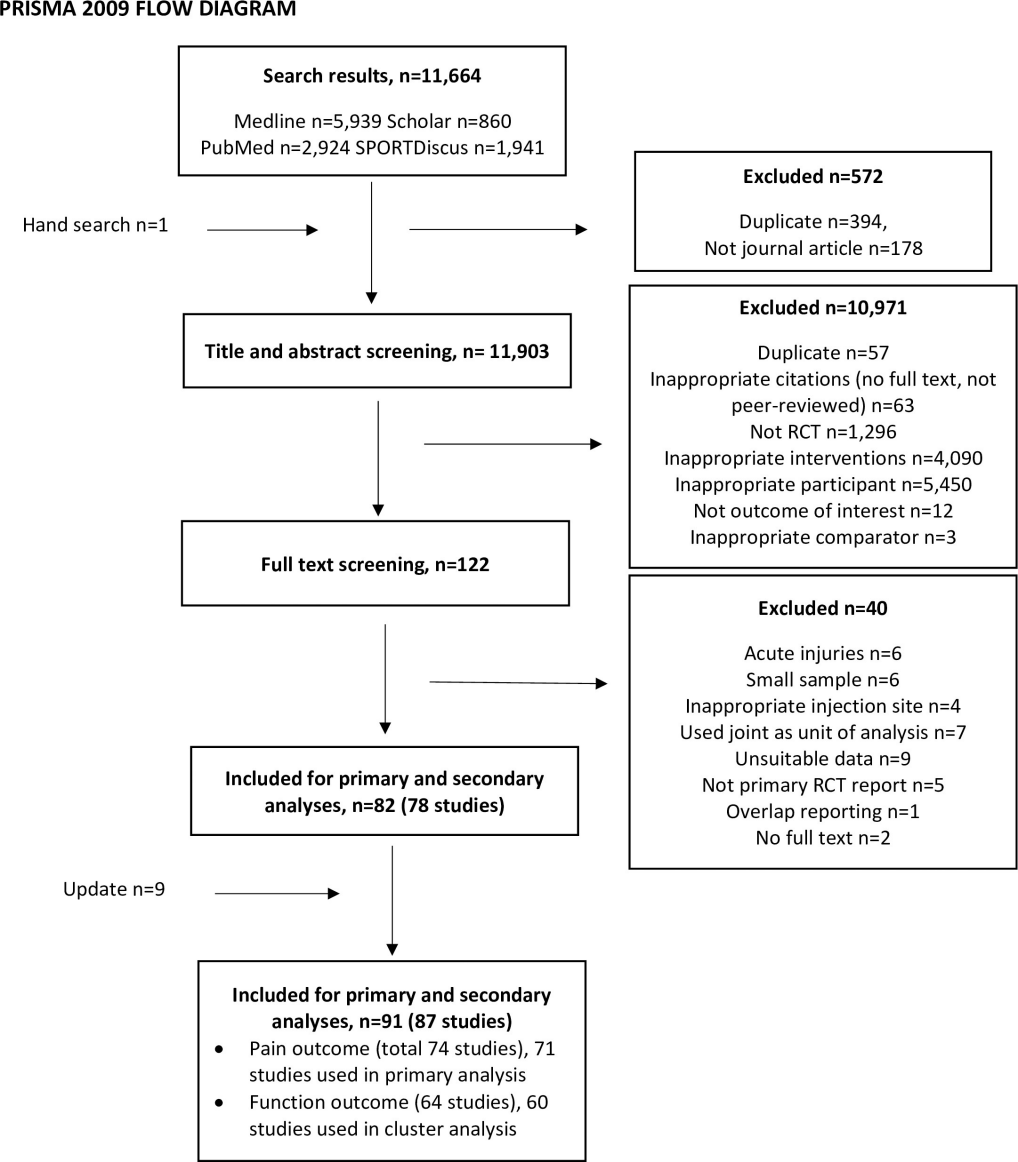
PRISMA flow chart.

A total of 11664 citations were identified from the initial search and yielded 78 studies for primary and secondary analyses for pain and/or functional outcomes (Fig 1). After the updated search, the included studies totalled 87. Cumulatively, these studies had 5859 participants - study level mean age ranged 31–52 years-old, male composition ranged 10–100%. Only four studies explicitly recruited patients who had not undergone any conservative treatment. In majority of studies (65 studies), the participants had to experience symptoms for period of 1–6 months before recruitment. The arm level mean duration of symptoms ranged 1 month to 15 years. Median follow-up time was six months (range: one week to three years). Types of CSTI being included were lateral/ medial epicondylitis (61%), tendinopathies of rotator cuffs (13%), Achilles tendon (8%), plantar fascia (8%), patella tendon (5%) and few other conditions in the truncal and pelvic regions. European countries (including United Kingdom) alone contributed the greatest number of studies (39%) followed by Asian countries (36%). Study design involving more than two treatment groups were found in 21 studies. Summary of the study characteristics and outcome measures reported are tabulated in S3 Table and S1 File, respectively.

### Pain outcome

**Fig 2:**
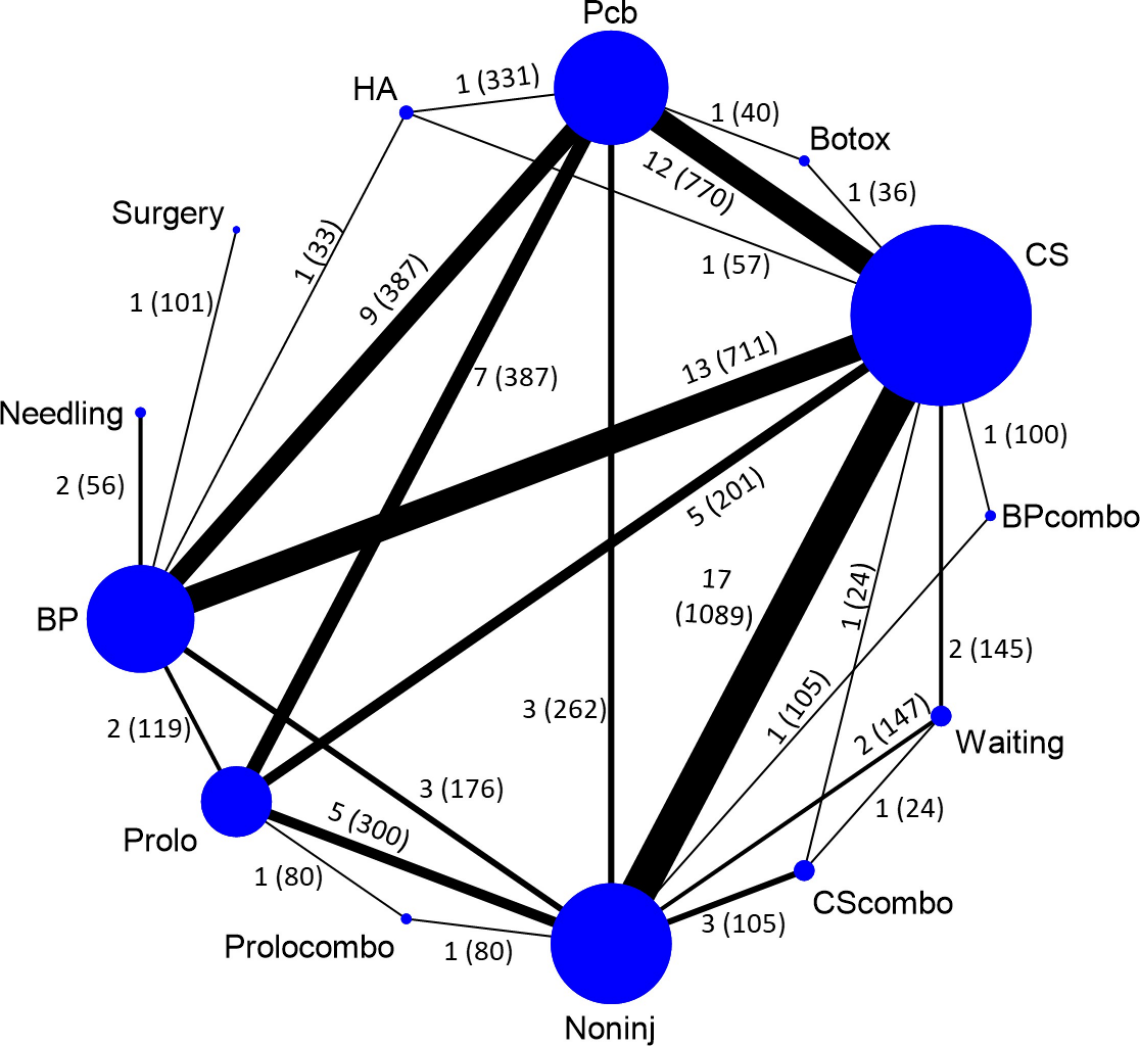
Network geometry. Note: BP = blood product; BPcombo = blood product combination therapy; Botox = botulinum toxin; CS = corticosteroid; CScombo = corticosteroid combination therapy; HA = hyaluronic acid; Noninj = non-injections; Pcb = placebo; Prolo = prolotherapy; Prolocombo = prolotherapy combination therapy. The size of the node corresponds to the number of participants assigned to the intervention while thickness of the connecting line corresponds to the number of the studies included.

Primary pain analysis was derived from 71 studies and secondary pain analysis from 74 studies. Although all competing treatments included from 71 studies formed a single connected network, many treatments (e.g. HA and needling) were connected by only a small number of studies (Fig 2). In the primary analysis, CS formed the largest group of injection in the network, widely being compared to other treatments. In contrast, the number of prolotherapy studies were relatively less and had only five pairs of head-to-head comparisons (i.e. Pcb, Noninj, CS, BP and Prolocombo). Time points included in the primary analysis averaged 4.5 months (ranged one week to 12 months) because many studies did not have long-term follow-up. There was no evidence of publication bias for placebo-controlled trials by visual inspection of funnel plot (S1 Fig).

### Relative efficacy

**Fig 3:**
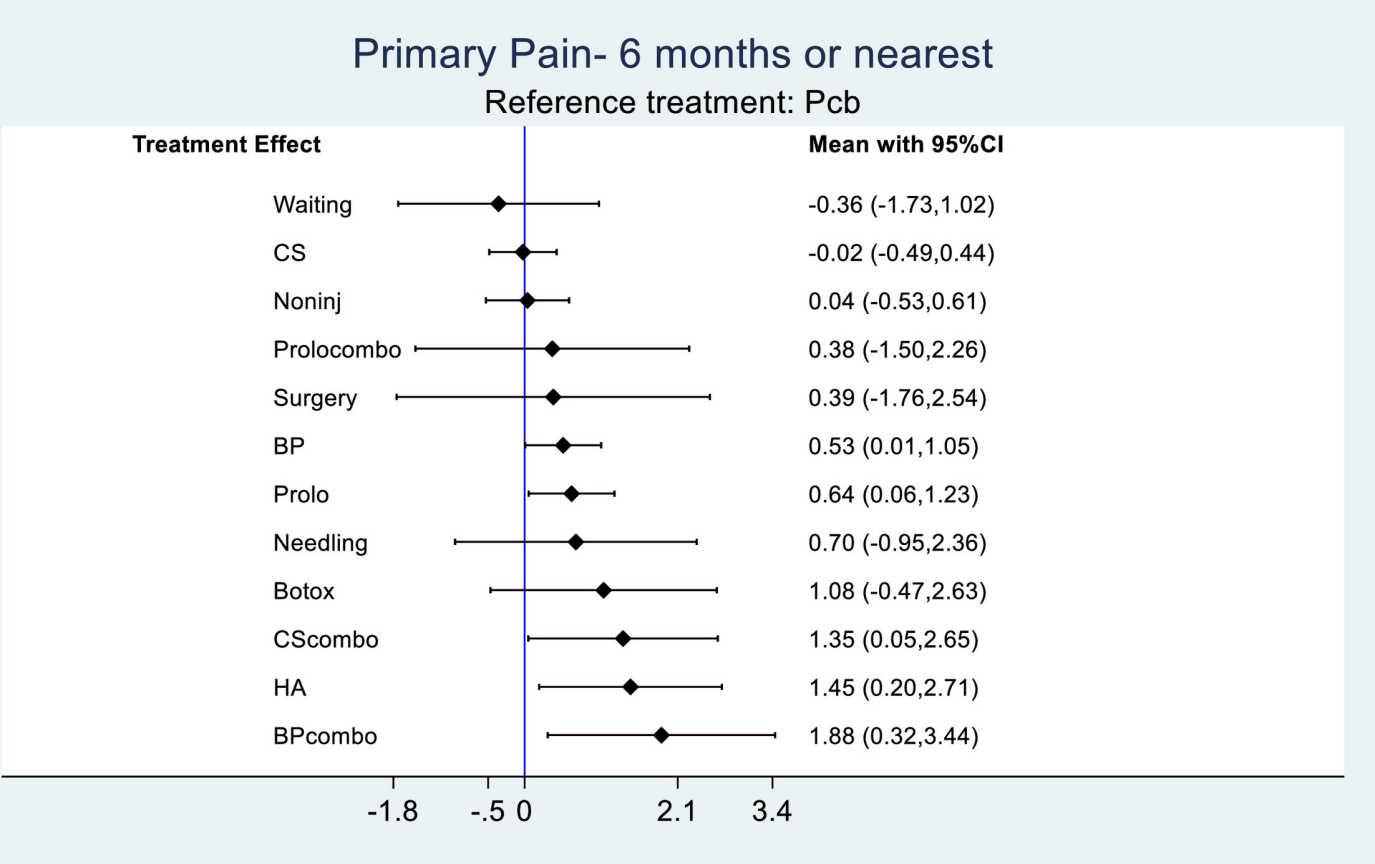
Interval plot of treatment efficacy relative to placebo. Note: BP = blood product; BPcombo = blood product combination therapy; Botox = botulinum toxin; CS = corticosteroid; CScombo = corticosteroid combination therapy; HA = hyaluronic acid; Noninj = non-injections; Pcb = placebo; Prolo = prolotherapy.

Given alone, prolotherapy was not as effective as CScombo, BPcombo and HA. Prolotherapy was better than CS and was equivalent to BP at six months (Fig 3). However, all three therapies (Prolo, CS and BP) did not reach statistical significance in pain improvement. There is no clear difference between injections types, and between injections and non-injections. When given as combination therapies, CS and BP provided relatively larger ES (ES CScombo 1.35; 95% CI 0.05 to 2.65; BPcombo 1.88; 95% CI 0.32 to 3.44) compared to when being used alone (ES CS -0.02; 95% CI -0.49 to 0.44; BP 0.53; 95% CI 0.01 to 1.05). The second largest ES (ES 1.45; 95% CI 0.20 to 2.71) after BPcombo was seen with HA. Although ESs for CScombo, HA and BPcombo were all significantly better than placebo, these estimates had relatively wide CIs suggesting substantial uncertainties.

### Quality of study

**Fig 4:**
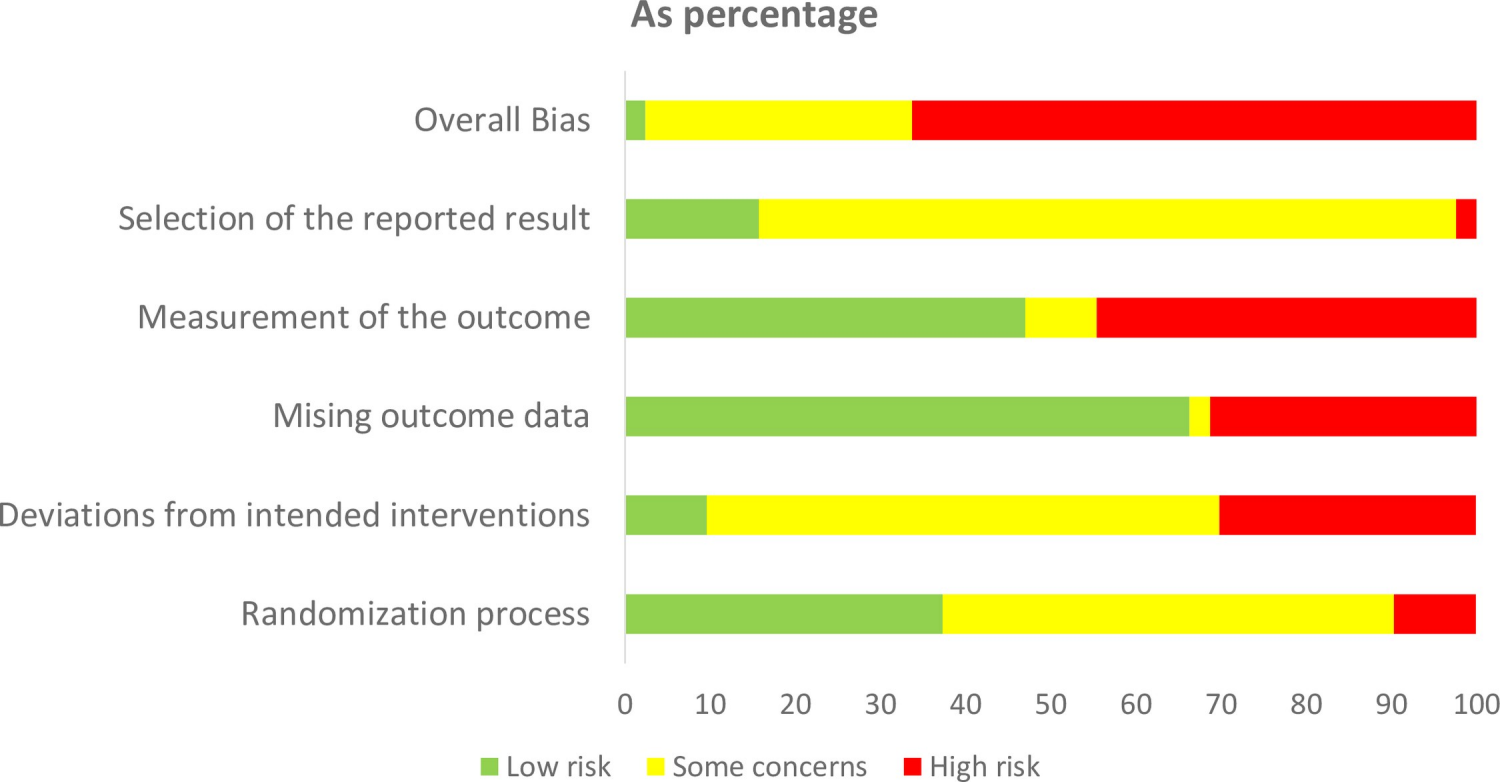
Risk of bias of individual study.

More than 60% of all the included studies, had at least one domain with high risk of bias (Fig 4). The domain related to measurement of outcome has the highest risk of bias due to the nature of self-reported outcomes being assessed by unblinded participants. GRADE assessment which was only performed for direct comparison between prolotherapy and other comparators (i.e. Pcb, CS, BP and Noninj) indicated the estimate for Prolo-CS and Prolo-BP to be of moderate strength while comparison between Prolo-Pcb and Prolo-Noninj to be very low and low strength, respectively (S4 Table).

### Sensitivity analysis

#### a. Change score

The direction of treatment effect for the interventions were relatively unchanged using change score (S2A Fig).

#### b. Time dependent analysis

*i*. *Overall*. As illustrated in S2Bi Fig. Prolo conferred consistent ES throughout all time points (<4 months: ES 0.47; 95% CI -0.07 to 1.02, 4–8 months: ES 0.50; 95% CI -0.48 to 1.47, >8 months: ES 0.46; 95% CI -0.28 to 1.20) and was consistently better than CS. Prolo appeared to be no different to BP at all time points, as evident by the closely overlapping CIs. For data points between 4–8 months post-intervention, none of the treatment groups (including all types of injection) were found to be better than placebo or wait-see policy (S2Bi Fig).

*ii*. *By region*. Rotator cuff tendinopathy and lateral epicondylitis were the most common conditions investigated at the shoulder and elbow region, respectively. Prolotherapy conferred significant pain relief earlier, albeit smaller effect, in shoulder injuries (ES <4 months: 0.65; 96% CI 0.00 to 1.30) in comparison to elbow injuries (ES 4–8 months: 0.91; 95% CI 0.12 to 1.70) (S2Bii and S2Biii Fig). Efficacy of prolotherapy beyond eight months was also different between shoulder and elbow injuries. While it conferred large and significant effect for shoulder injuries (ES 2.08; 95% CI 1.49 to 2.68), it was no better than placebo for elbow injuries (ES -0.21; 95% CI-1.75 to 1.34). Estimates for shoulder beyond eight months were derived from only three studies and those of elbow from 13 studies.

In contrast to prolotherapy, CS demonstrated a regression in treatment effect for both elbow and shoulder injuries across time.

*iii*. *Study quality*. Excluding studies with high risk of bias in randomization did not alter the conclusion of the primary analysis (S2C Fig). Excluding studies with high risk of bias in missing outcomes data, however, reduced the magnitude of ES for botox (ES 1.08; 95% CI -0.47 to 2.63 to ES 0.05; 95% CI -1.91 to 2.02) and HA (ES 1.45; 95% CI 0.20 to 2.71 to ES 0.88; 95% CI -0.62 to 2.33), rendering HA no longer significantly better than placebo. Following exclusion of studies with high risk of bias in missing data, the ES for prolotherapy also reduced, but with more uncertainty around the point estimate, - from 0.64 (95% CI -0.06 to 1.23) to 0.36 (95% CI -1.38 to 2.11).

### Functional outcome

**Fig 5:**
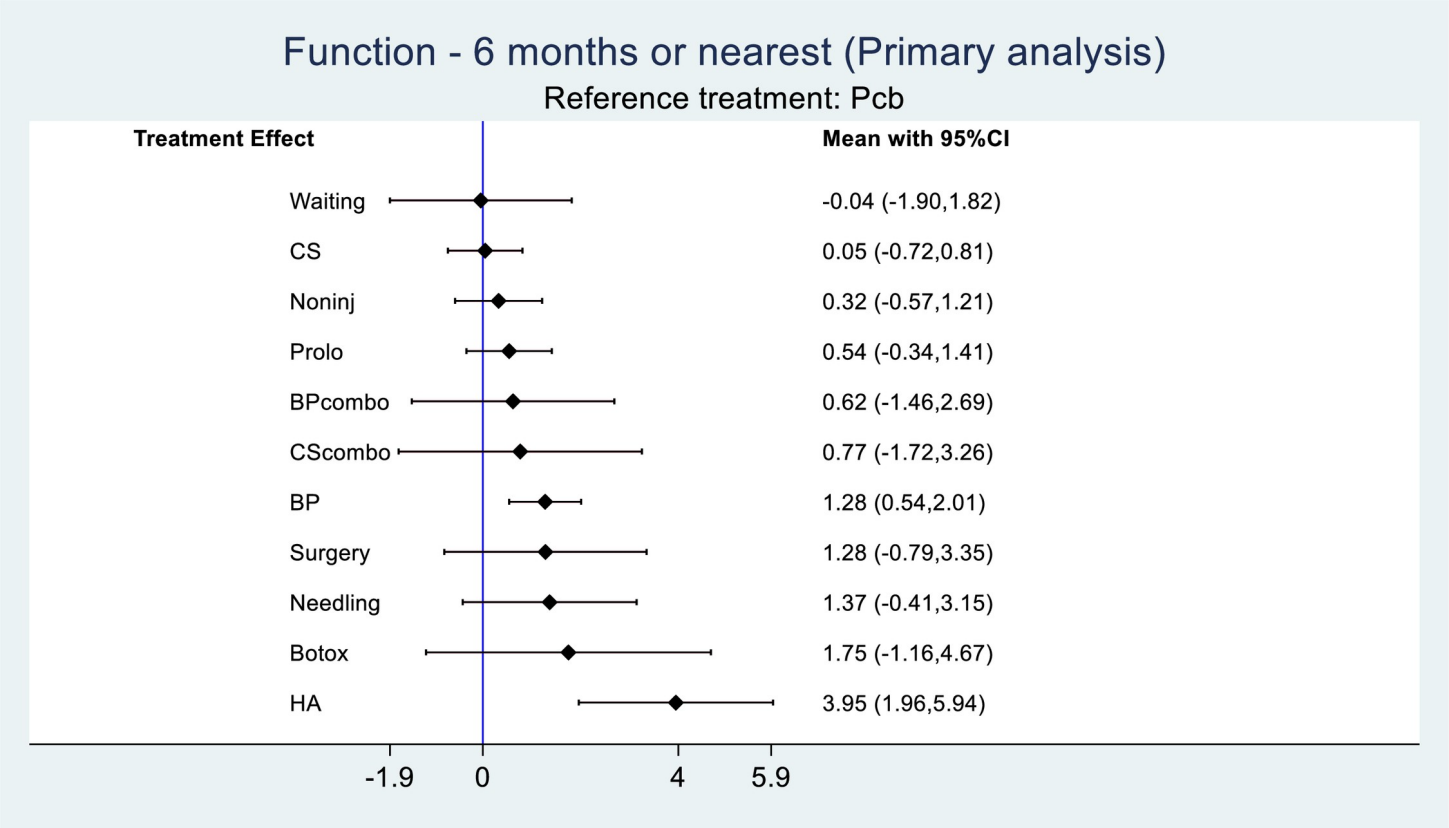
Functional outcome. Note: BP = blood product; BPcombo = blood product combination therapy; Botox = botulinum toxin; CS = corticosteroid; CScombo = corticosteroid combination therapy; HA = hyaluronic acid; Noninj = non-injections; Pcb = placebo; Prolo = prolotherapy.

Functional outcome analysis was derived from 60 studies. Functional outcome was significantly better with BP and HA, but not with prolotherapy or other therapies (Fig 5). BP, surgery and needling appear to produce greater benefit for functional outcome (ES>1) than for pain outcome (ES<1). For prolotherapy, the magnitude of functional improvement was matched by the improvement in pain.

### Secondary analysis

Prolotherapy was as effective as all preparations of PRP (S3–1 Fig). As a group, Inj (i.e. Prolo, BP, CS PRP and HA) (ES 0.19; 95% CI -0.66 to 1.04) were not significantly better than Noninj (ES 0.11; 95% CI -0.78 to 0.99) (S3–2 Fig). Placebo effect were similar irrespective of whether it was delivered in injection or non-injection form.

### Cluster analysis

Relative efficacy of prolotherapy for functional outcome showed that Prolo, BPcombo, HA and Noninj were in the same cluster being more effective for pain than for function outcome (S4 Fig).

### Validation

The direct and indirect estimates for BP-Pcb, BP-CS and Noninj-Prolo were found to be statistically different in side splitting method (S5A Fig). However, the inconsistency in evidence loop was only evident for BP-Pcb and BP-CS comparison (S5B Fig). In the presence of these local inconsistency in the network, the results were further explored using inconsistency model. The estimated ES for prolotherapy was reduced by 0.2 in the inconsistency model (ES for the consistency model 0.64; 95% CI 0.06 to 1.23; inconsistency model: 0.44; 95% CI -2.03 to 2.91) being less efficacious than BP, HA and Botox (S5C Fig). No treatment appeared to be significantly better than placebo in the consistency or the inconsistency model.

In the primary analysis with overall studies, the between studies SD is large 1.04. In subgroup analyses (i.e. by time, region and study quality) RoB, the values of between studies SD generally remained large ranging from 0.7 to 0.9. Exception was seen for analyses of elbow at 4–8 months and of shoulder at <4 months, for which the between study SD was 0.4 and 0.5, respectively.

## Discussion

This NMA aimed to compare the efficacy of prolotherapy and other commonly used therapies for reducing pain in CSTI. Based on 87 RCTs, this study demonstrated how the magnitude and order of treatment effect changed with different clinical subgrouping (i.e. time point measures, and injury site), but also with different underlying assumptions in NMA (i.e. consistent and inconsistent modelling) and study quality (i.e. risk of bias). Overall, the results suggested that all therapies (except HA) were not significantly better than placebo, irrespective of time point and injury type. Prolotherapy was significantly better than placebo in elbow injuries in the medium term (4–8 months) and in shoulder injuries in the short term (<4 months) and long term (>8 months). Although the ES of prolotherapy was not greater than other injections, it was generally better than Noninj and CS for improving symptoms and function.

Unlike some NMA that focused on a specific injury [18, 19], or limited their analysis to only few injections [20], this study included a wide range of conditions and injection therapies, allowing the efficacy of prolotherapy for CSTI to be generalised. Nevertheless, the efficacy of prolotherapy for specific injuries (i.e. rotator cuff injuries and lateral epicondylitis) could still be estimated separately using subgroup analysis. For more comprehensive evaluation, additional analyses were undertaken to understand the effect of different methods of treatment grouping on the results obtained. The advantage of this approach is that the comparison between different injuries, between different time points and different grouping approaches can be performed cohesively within a single NMA.

The NMA for functional outcomes was performed to explore if improvement of symptoms could be translated to functional improvement. Overall, there was no definitive link between pain and functional improvement in CSTI, which could be attributed to the fact that not all RCTs assessed functional outcomes alongside pain outcomes and vice versa. Also, the presence of mediating factors such as patient characteristics [21] and the use of non-standard outcome measurement tools could also dilute the relationship between pain and function [22].

Although non-injection therapies were not homogeneous and should arguably be represented as separate nodes in the network, we had intended for all non-injection therapies to be analysed as a single group. This is because the group effect of non-injection therapies is typically considered in clinical setting before patient-doctor make a shared decision to move up the treatment ladder. Furthermore, published guidelines recommend injection therapies to be given only after multiple non-injection therapies have failed [23, 24]. Therefore, the non-injection therapies were aptly represented as a single node in this NMA.

A notable observation from our primary, secondary and subgroup analyses was the large and significant effect conferred by HA. HA is widely believed to combat chronic and degenerative articular cartilage injury through various pathways, including controlling inflammation and promoting regenerative processes [25]. For this reason, the utility of HA has now expanded to treatment of CSTI [26] where primary pathology involves poor tissue healing [27]. However, the large ES for HA reported in this study should not be regarded as definitive evidence of efficacy as it was derived from only three RCTs and the wide CI suggested high uncertainty.

The estimate for HA in shoulder injuries was not consistent with the small effect found in a recent NMA which used different inclusion criteria [28]. The RCTs where injections were delivered into subacromial bursa or other clinical conditions without specific treatment target (e.g. subacromial impingement) were included in the NMA by Lin et al but excluded in this NMA. Despite methodological differences, the findings that the analgesic effect of CS was short-lived and that there was no clear difference between prolotherapy and other injection therapies were consistent with that of other investigators [18].

Considering the results of primary and secondary analyses, it would be reasonable to infer that there is generally no clear difference between prolotherapy and other injection therapies. Nevertheless, there is a trend suggesting that combination therapy could offer additional benefits, irrespective of the injection agents used. This result is consistent with the injury continuum theory which suggests the existence of multiple treatment targets for CSTI [4, 29], implicating the importance of proper patient selection, and sound understanding of the underlying pathology and pain mechanism when individualising injection therapies.

These inferences need to be interpreted cautiously as the results were shown to be sensitive to measurement time point and missing outcome data. Furthermore, more than half of the included studies had high overall risk of bias and some pairs of comparison were connected by small number of RCTs. Another caveat for this NMA is the presence of significant inconsistency between direct and indirect estimates for BP-Pcb, BP-CS and Noninj-Prolo comparisons. However, no clear underlying explanation could be found. Therefore, subgroup and post-hoc analyses based on inconsistency modelling was done to explore the extent of its effect on the results. Results from inconsistency model produced wider estimates and changed the magnitude of ES for prolotherapy, but still consistently demonstrated no significant difference between prolotherapy and other injections. The modifying/ mediating effects of some clinical (e.g. injury severity, age, gender) and study characteristics (e.g. drug concentration, delivery frequency, constituents of injection), were not accounted for in this analysis as these were not related to the primary aim and would be best evaluated using separate data collection and analysis.

## Supporting information

S1 Table PRISMA NMA checklist. (DOCX)

S2 Table Search strategy (Medline). (DOCX)

S3 Table Study characteristics. # Multiple reports * Treatment groups were aggregated based on the injection therapy for analysis. Note: ESWT = Extracorporeal shock wave therapy; mths = months; PRP = platelet rich plasma; Prolo = prolotherapy; Rpt = repeat; Y = yes.
(DOCX)

S4 Table GRADE assessment of RCTs performing direct comparisons between prolotherapy and other therapies. (DOCX)

S1 Fig Funnel plot for placebo-controlled trials. A = blood product; C = botulinum toxin; D = corticosteroid, F = hyaluronic acid; H = non-injections; I = placebo.
(DOCX)

S2 Fig Sensitivity and subgroup analysis by change score, time points, region and study bias. Note: BP = blood product; BPcombo = blood product combination therapy; Botox = botulinum toxin; CS = corticosteroid; CScombo = corticosteroid combination therapy; HA = hyaluronic acid; Noninj = non-injections; Pcb = placebo; Prolo = prolotherapy.
(DOCX)

S3 Fig Secondary analysis (alternative treatment grouping). (DOCX)

S4 Fig Cluster analysis. Note: BP = blood product; BPcombo = blood product combination therapy; Botox = botulinum toxin; CS = corticosteroid; CScombo = corticosteroid combination therapy; HA = hyaluronic acid; Noninj = non-injections; Pcb = placebo; Prolo = prolotherapy.
(DOCX)

S5 Fig Validation: Network sidesplit, Forest plot by design and Inconsistency model of primary analysis. A. Network sidesplit. *The results are derived from direct evidence only. Note: A = blood product; B = blood product combination therapy; C = botulinum toxin; D = corticosteroid; E = corticosteroid combination therapy; F = hyaluronic acid; G = needling; H = non-injections; I = placebo; J = prolotherapy; K = surgery; L = wait-see policy; Yellow = p<0.05 indicating significant difference between direct and indirect estimates. B. Forest plot by design. Note: BP = blood product; BPcombo = blood product combination therapy; Botox = botulinum toxin; CS = corticosteroid; CScombo = corticosteroid combination therapy; HA = hyaluronic acid; Noninj = non-injections; Pcb = placebo; Prolo = prolotherapy. Yellow: Direct estimate from A-D-I (blood product—corticosteroid—placebo) studies did not overlap with the overall estimates for placebo-blood product comparison. Direct estimates from A-D (blood product—corticosteroid) studies did not overlap with the estimates from A-D-I studies and from overall. C. Inconsistency model for primary analysis. Note: BP = blood product; BPcombo = blood product combination therapy; Botox = botulinum toxin; CS = corticosteroid; CScombo = corticosteroid combination therapy; HA = hyaluronic acid; Noninj = non-injections; Pcb = placebo; Prolo = prolotherapy.
(DOCX)

S1 File Outcome measures. (XLSX)

## References

[1] DiFiori JP, Benjamin HJ, Brenner JS, Gregory A, Jayanthi N, Landry GL, et al. Overuse injuries and burnout in youth sports: a position statement from the American Medical Society for Sports Medicine. Br J Sports Med. 2014 2 1;48(4):287–8. 10.1136/bjsports-2013-093299 24463910

[2] Steinmann S, Pfeifer CG, Brochhausen C, Docheva D. Spectrum of tendon pathologies: triggers, trails and end-state. International Journal of Molecular Sciences. 2020 1;21(3):844. 10.3390/ijms21030844 32013018 PMC7037288

[3] Brukner P, Bennell K. Overuse injuries: where to now? Br J Sports Med. 1997 3;31(1):2. 10.1136/bjsm.31.1.2 9132203 PMC1332463

[4] Cook JL, Rio E, Purdam CR, Docking SI. Revisiting the continuum model of tendon pathology: what is its merit in clinical practice and research? Br J Sports Med. 2016 10 1;50(19):1187–91. 10.1136/bjsports-2015-095422 27127294 PMC5118437

[5] Docking SI, Rio E, Cook J, Carey D, Fortington L. Quantification of Achilles and patellar tendon structure on imaging does not enhance ability to predict self-reported symptoms beyond grey-scale ultrasound and previous history. J Sci Med Sport. 2019 2 1;22(2):145–50. 10.1016/j.jsams.2018.07.016 30098975

[6] Bhabra G, Wang A, Ebert JR, Edwards P, Zheng M, Zheng MH. Lateral elbow tendinopathy: development of a pathophysiology-based treatment algorithm. Orthop J Sports Med. 2016 11 1;4(11):2325967116670635. 10.1177/2325967116670635 27833925 PMC5094303

[7] Corrigan P, Cortes DH, Pohlig RT, Grävare Silbernagel K. Tendon morphology and mechanical properties are associated with the recovery of symptoms and function in patients with Achilles tendinopathy. Orthop J Sports Med. 2020 4 30;8(4):2325967120917271. 10.1177/2325967120917271 32426410 PMC7218994

[8] Hauser RA, Lackner JB, Steilen-Matias D, Harris DK. A Systematic Review of Dextrose Prolotherapy for Chronic Musculoskeletal Pain. Clin Med Insights Arthritis Musculoskelet Disord. 2016;9:.S39160–21. 10.4137/CMAMD.S39160 27429562 PMC4938120

[9] Goswami A. Prolotherapy. J Pain Palliat Care Pharmacother. 2012 12 5;26(4):376–8. 10.3109/15360288.2012.734900 23216178

[10] Lee JC, Ahmed N, Allen GM. Image guided injection therapies in athletes—Do they work and what should we be using? Eur J Radiol. 2019;110:193–202. 10.1016/j.ejrad.2018.12.001 30599860

[11] Di Matteo B, Filardo G, Kon E, Marcacci M. Platelet-rich plasma: evidence for the treatment of patellar and Achilles tendinopathy—a systematic review. Musculoskelet Surg. 2015 4 1;99(1):1–9. 10.1007/s12306-014-0340-1 25323041

[12] Kaux JF, Samson A, Crielaard JM. Hyaluronic acid and tendon lesions. Muscles Ligaments Tendons J. 2015 10;5(4):264. 10.11138/mltj/2015.5.4.264 26958533 PMC4762636

[13] Aicale R, Bisaccia RD, Oliviero A, Oliva F, Maffulli N. Current pharmacological approaches to the treatment of tendinopathy. Expert Opin Pharmacother. 2020 6 7:1–1. 10.1080/14656566.2020.1763306 32511031

[14] Wan X, Wang W, Liu J, Tong T. Estimating the sample mean and standard deviation from the sample size, median, range and/or interquartile range. BMC Med Res Methodol. 2014 12 1;14(1):135. 10.1186/1471-2288-14-135 25524443 PMC4383202

[15] Sterne JA, Savović J, Page MJ, Elbers RG, Blencowe NS, Boutron I, et al. RoB 2: a revised tool for assessing risk of bias in randomised trials. BMJ. 2019 8 28;366. 10.1136/bmj.l4898 31462531

[16] Guyatt G, Oxman AD, Akl EA, Kunz R, Vist G, Brozek J, et al. GRADE guidelines: 1. Introduction—GRADE evidence profiles and summary of findings tables. J. Clin. Epidemiol. 2011 4 1;64(4):383–94. 10.1016/j.jclinepi.2010.04.026 21195583

[17] Bannuru RR, Schmid CH, Kent DM, Vaysbrot EE, Wong JB, McAlindon TE. Comparative effectiveness of pharmacologic interventions for knee osteoarthritis: a systematic review and network meta-analysis. Ann Intern Med. 2015 1 6;162(1):46–54. 10.7326/M14-1231 25560713

[18] Lian J, Mohamadi A, Chan JJ, Hanna P, Hemmati D, Lechtig A, et al. Comparative efficacy and safety of nonsurgical treatment options for enthesopathy of the extensor carpi radialis brevis: a systematic review and meta-analysis of randomized placebo-controlled trials. Am J Sports Med. 2019 10;47(12):3019–29. 10.1177/0363546518801914 30380334

[19] Tsikopoulos K, Vasiliadis HS, Mavridis D. Injection therapies for plantar fasciopathy (‘plantar fasciitis’): a systematic review and network meta-analysis of 22 randomised controlled trials. Br J Sports Med. 2016 11 1;50(22):1367–75. 10.1136/bjsports-2015-095437 27143138

[20] Tang S, Wang X, Wu P, Wu P, Yang J, Du Z, et al. Platelet‐Rich Plasma Vs Autologous Blood Vs Corticosteroid Injections in the Treatment of Lateral Epicondylitis: A Systematic Review, Pairwise and Network Meta‐Analysis of Randomized Controlled Trials. PM&R. 2020 4;12(4):397–409. 10.1002/pmrj.12287 31736257 PMC7187193

[21] Steiner JL, Bigatti SM, Slaven JE, Ang DC. The complex relationship between pain intensity and physical functioning in fibromyalgia: the mediating role of depression. J Appl Biobehav Res. 2017 12;22(4):e12079. 10.1111/jabr.12079 29527113 PMC5839337

[22] Sadosky A, Parsons B, Emir B, Nieshoff EC. Pain relief and functional improvement in patients with neuropathic pain associated with spinal cord injury: an exploratory analysis of pregabalin clinical trials. J Pain Res. 2016;9:405. 10.2147/JPR.S97770 27366103 PMC4913987

[23] Doiron-Cadrin P, Lafrance S, Saulnier M, Cournoyer É, Roy JS, Dyer JO, et al. Shoulder rotator cuff disorders: a systematic review of clinical practice guidelines and semantic analyses of recommendations. Arch Phys Med Rehabil.2020 1 31. 10.1016/j.apmr.2019.12.017 32007452

[24] Figueroa D, Figueroa F, Calvo R. Patellar tendinopathy: diagnosis and treatment. J Am Acad Orthop Surg. 2016 12 1;24(12): e184–92. 10.5435/JAAOS-D-15-00703 27855131

[25] Altman RD, Manjoo A, Fierlinger A, Niazi F, Nicholls M. The mechanism of action for hyaluronic acid treatment in the osteoarthritic knee: a systematic review. BMC Musculoskelet Disord. 2015 12;16(1):1–0. 10.1186/s12891-015-0775-z 26503103 PMC4621876

[26] Fogli M, Giordan N, Mazzoni G. Efficacy and safety of hyaluronic acid (500–730kDa) Ultrasound-guided injections on painful tendinopathies: a prospective, open label, clinical study. Muscles Ligaments Tendons J. 2017 4;7(2):388. 10.11138/mltj/2017.7.2.388 29264351 PMC5725189

[27] Aicale R, Tarantino D, Maffulli N. Overuse injuries in sport: a comprehensive overview. J Orthop Surg Res. 2018 12;13(1):1–1. 10.1186/s13018-017-0693-x 30518382 PMC6282309

[28] Lin MT, Chiang CF, Wu CH, Huang YT, Tu YK, Wang TG. Comparative effectiveness of injection therapies in rotator cuff tendinopathy: a systematic review, pairwise and network meta-analysis of randomized controlled trials. Arch Phys Med Rehabil. 2019 2 1;100(2):336–49. 10.1016/j.apmr.2018.06.028 30076801

[29] Sudoł-Szopińska I, Kwiatkowska B, Prochorec-Sobieszek M, Maśliński W. Enthesopathies and enthesitis. Part 1. Etiopathogenesis. J Ultrason. 2015 3;15(60):72. 10.15557/JoU.2015.0006 26674568 PMC4579704

