# Supplementary material for: Efficacy of prolotherapy in comparison to other therapies for chronic soft tissue injuries: A systematic review and network meta-analysis
Source: PLoS One. 2021 May 26;16(5):e0252204. doi: 10.1371/journal.pone.0252204 (PMC8153441; doi:10.1371/journal.pone.0252204)
Supplement: S2 Table — (DOCX) [file pone.0252204.s002.docx]

**S2 Table. Search strategy (Medline)**

| S1 | AB prolotherapy |
| --- | --- |
| S2 | (MH "Prolotherapy") OR "prolotherapy" |
| S3 | AB prolothera* OR proliferat* |
| S4 | AB ((hypertonic adj5 dextrose) or (hypertonic adj5 glucose) or phenolglucose-glycerine or p2g or gluco* or dextro* or tannic acid or guaiacol or guaiakol or phenol or glycerin* or zinc sulfat*) |
| S5 | AB sclerosing* or sclerothera* or sclero-thera* |
| S6 | AB glucose or dextrose or morrhuate or phenol |
| S7 | S1 OR S2 OR S3 OR S4 OR S5 OR S6 |
| S8 | PT randomized controlled trial |
| S9 | PT controlled clinical trial |
| S10 | PT controlled clinical trial |
| S11 | AB random$ OR AB trial$ OR AB RCT |
| S12 | S8 OR S9 OR S10 OR S11 |
| S13 | (S7 AND S12) NOT animal |
| S14 | ( (S7 AND S12) NOT animal ) AND AB ( muscle* OR sprain* OR strain* OR injur* OR tend* OR ligament* ) |
