# Supplementary material for: Efficacy of prolotherapy in comparison to other therapies for chronic soft tissue injuries: A systematic review and network meta-analysis
Source: PLoS One. 2021 May 26;16(5):e0252204. doi: 10.1371/journal.pone.0252204 (PMC8153441; doi:10.1371/journal.pone.0252204)
Supplement: S3 Table — # Multiple reports * Treatment groups were aggregated based on the injection therapy for analysis. Note: ESWT = Extracorporeal shock wave therapy; mths = months; PRP = platelet rich plasma; Prolo = prolotherapy; Rpt = repeat; Y = yes. (DOCX) [file pone.0252204.s003.docx]

**S3 Table. Study Characteristics**

| Author, Year (Country) | Diagnosis/Imaging/ minimum symptoms | N subject (% men); mean age (SD or range) | Outcome (s) | G1 | | G2 | | G3 | | G4 | |
| --- | --- | --- | --- | --- | --- | --- | --- | --- | --- | --- | --- |
|  |  |  |  | Injectable | Non-Injectable | Injectable | Non-Injectable | Injectable | Non-Injectable | Injectable | Non-Injectable |
| Ahadi, 2019 (Iran) | Lateral epicondylitis/ Y/ 3 mths | 33(30.3); - | Pain, Function | Dextrose 20%, Lignocaine --Rpt: (x) | -- | ESWT, | -- | -- | -- | -- | -- |
| Ahmed, 2012 (India) | Lateral epicondylitis/ -/ 3 mths | 60(75); - | Pain | Triamcinolone, Lignocaine --Rpt: - | NSAIDs, | -- | NSAIDs, | -- | -- | -- | -- |
| Akcay, 2020 (Turkey) | Lateral epicondylitis/ -/ 3 mths | 50(26); - | 50(26); - | Prolo, --Rpt: - | Exercise, | Saline, --Rpt: - | Exercise, | -- | -- | -- | -- |
| Alvarez, 2005 (Canada) | Rotator cuff tendinopathy or partial tears/ Y/ 6 mths | 62(NA); - | Pain, Function | Bethamethasone, Lignocaine --Rpt: - | Lignocaine, --Rpt: (x) | -- | -- | -- | -- | -- | -- |
| Arik, 2014 (Turkey) | Lateral epicondylitis/ -/ - mths | 80(26.25); - | Pain, Function | Whole blood, Prilocaine --Rpt: - | Methyl Prednisolone, Prilocaine --Rpt: - | -- | -- | -- | -- | -- | -- |
| Asheghan, 2019 (Iran) | Plantar fasciitis/ -/ 2 mths | 59(33.8983050847458); 45(6.9) | 59(33.8983050847458); 45(6.9) | Prolo, --Rpt: Weekly (2x) | -- | Electomodalities, | -- | -- | -- | -- | -- |
| Bayat, 2019 (Iran) | Lateral epicondylitis/ -/ 3 mths | 28(39); - | Pain, Function | Methyl Prednisolone, Lignocaine --Rpt: - | Exercise, | Prolo, --Rpt: - | Exercise, | -- | -- | -- | -- |
| Behera, 2015 (India) | Lateral epicondylitis/ -/ 3 mths | 25(NA); - | Pain, Function | PRP (unclear), --Rpt: - | Exercise, | Marcaine/Bupivacaine, --Rpt: - | Exercise, | -- | -- | -- | -- |
| Bell, 2013 (New Zealand) | Midportion Achilles tendinopathy/ Y/ 3 mths | 53(52.8); - | Function | Whole blood, --Rpt: Monthly (2x) | Exercise, | Needling, --Rpt: Monthly (2x) | Exercise, | -- | -- | -- | -- |
| Bertrand, 2016 (Canada) | Rotator cuff tendinopathy/ Y/ 3 mths | 77(NA); 51(-) | Pain | Dextrose 25%, Lignocaine --Rpt: Monthly (3x) | physiotherapy, | Saline, --Rpt: Monthly (3x) | physiotherapy, | Sham injection, --Rpt: Monthly (3x) | physiotherapy, | -- | -- |
| Boesen, 2017 (Denmark) | Midportion Achilles tendinopathy/ Y/ 3 mths | 57(100); - | Pain | Methyl Prednisolone, Marcaine/Bupivacaine --Rpt: - | Exercise, | PRP (unclear), --Rpt: Fortnightly (4x) | Exercise, | Saline, --Rpt: Fortnightly (4x) | Exercise | -- | -- |
| Branson, 2017 (Australia) | Lateral epicondylitis/ Y/ 2 mths | 44(63.6); 48(7.5) | Function | Bethamethasone, --Rpt: - | Whole blood, --Rpt: Variable (2x) | Polidocanol, --Rpt: Variable (2x) | -- | -- | -- | -- | -- |
| Carayannopoulos, 201 (US) | Lateral epicondylitis/ -/ 3 mths | 17(35); 46(35-57) | Pain, Function | Prolo, procaine --Rpt: Monthly (2x) | Methyl Prednisolone, --Rpt: - |  | -- | -- | -- | -- | -- |
| Cole, 2018 (Australia) | supraspinatus tendinopathy/ Y/ 3 mths | 36(75); 48(22-78) | Pain | Dextrose 25%, Lignocaine --Rpt: - | physiotherapy, | Methyl Prednisolone, Lignocaine --Rpt: - | physiotherapy, | -- | -- | -- | -- |
| Creaney, 2011 (UK) | Lateral epicondylitis/ -/ 6 mths | 150(NA); - | Function | PRP (unclear), --Rpt: Monthly (2x) | Whole blood, --Rpt: Monthly (2x) |  | -- | -- | -- | -- | -- |
| Crowther, 2002 (UK) | lateral epicondylitis/ -/ 4 mths | 93(NA); 49(27-69) | Pain | Triamcinolone, Lignocaine --Rpt: - | -- | ESWT, | -- | -- | -- | -- | -- |
| Damjanov, 2018 (Serbia) | Supraspinatus tendinopathy/ Y/ 1.5 mths | 32(34.375); - | Pain | ACP, --Rpt: Weekly (4x) | Bethamethasone, --Rpt: Weekly (3x) |  | -- | -- | -- | -- | -- |
| de Vos, 2010 (Netherland) | Achilles tendinopathy/ -/ 2 mths | 54(NA); - | Function | PRP (unclear), Marcaine/Bupivacaine --Rpt: - | Exercise, | Saline, --Rpt: - | Exercise, | -- | -- | -- | -- |
| Dogramaci, 2009 (Turkey) | lateral epicondylitis/ -/ - mths | 75(NA); - | Pain | Triamcinolone, Lignocaine --Rpt: as needed | Triamcinolone, Lignocaine --Rpt: as needed | Lignocaine, --Rpt: as needed | -- | -- | -- | -- | -- |
| Dragoo, 2014 (US) | patella tendinopathy/ Y/ 1.5 mths | 21(95); 35(13) | Pain, Function | Leucocyte rich PRP, Marcaine/Bupivacaine --Rpt: - | Exercise, | Needling, Marcaine/Bupivacaine --Rpt: - | Exercise, | -- | -- | -- | -- |
| Ebbesen, 2018 (Denmark) | Achilles tendon/ Y/ 3 mths | 48(NA); - | Pain, Function | Polidocanol, --Rpt: Monthly (2x) | Lignocaine, --Rpt: (x) | -- | -- | -- | -- | -- | -- |
| Elizondo-Rodriguez, 2013 (Mexico) | Plantar Fasciitis/ -/ 3 mths | 40(NA); - | Pain, Function | Botulinum toxin, --Rpt: - | Exercise, | Dexamethasone, Lignocaine --Rpt: - | Exercise, | -- | -- | -- | -- |
| Ersen, 2018 (Turkey) | Plantar Fasciitis/ -/ - mths | 60(NA); - | Pain, Function | Dextrose 15%, Lignocaine --Rpt: Weekly (3x) | -- | Exercise, | -- | -- | -- | -- | -- |
| Fitzpatrick, 2018 (Australia) | Gluteal tendinopathy/ Y/ 4 mths | 80(10); - | Function | PRP (unclear), Lignocaine --Rpt: - | physiotherapy, | Bethamethasone, Lignocaine --Rpt: - | physiotherapy, | -- | -- | -- | -- |
| Gautam, 2015 (India) | Lateral epicondylitis/ Y/ 6 mths | 30(NA); - | Pain, Function | PRP (unclear), --Rpt: - | Methyl Prednisolone, --Rpt: - |  | -- | -- | -- | -- | -- |
| Goyal, 2019 (India) | lateral epicondylitis/ -/ - mths | 60(NA); 31.11(20-40) | Function | PRP (unclear), --Rpt: - | physiotherapy, | Triamcinolone, --Rpt: - | physiotherapy, | -- | -- | -- | -- |
| Gupta, 2020 (India) | Lateral epicondylitis/ -/ mths | 80(42.5); 40 (-) | 80(42.5); 40 (-) | PRP (unclear), --Rpt: - | Triamcinolone, --Rpt: - |  | -- | -- | -- | -- | -- |
| Hart, 2002 (Netherland) | Lateral epicondylitis/ -/ 1.5 mths | 185(NA); - | Pain, Function | Triamcinolone, Lignocaine --Rpt: Variable (3x) | -- | physiotherapy, | -- | Wait-see policy, | -- | -- | -- |
| Hay, 1999 (UK) | Lateral epicondylitis/ -/ - mths | 164(18.9); - | Pain, Function | Methyl Prednisolone, Lignocaine --Rpt: - | -- | NSAIDs, | -- | Sham/placebo, | -- | -- | -- |
| Hayton, 2005 (UK) | Lateral epicondylitis/ -/ 6 mths | 40(52.5); 48(35-71) | Pain | Botulinum toxin, --Rpt: - | Saline, --Rpt: Variable (2x) | -- | -- | -- | -- | -- | -- |
| Jensen, 2001 (Denmark) | Lateral epicondylitis/ -/ - mths | 30(43); 46(28-60) | Pain | Methyl Prednisolone, Lignocaine --Rpt: as needed | -- | -- | Orthosis | -- | -- | -- | -- |
| Jindal, 2013 (India) | lateral epicondylitis/ -/ - mths | 50(NA); - | Pain, Function | Methyl Prednisolone, Lignocaine --Rpt: - | Whole blood, Lignocaine --Rpt: - | -- | -- | -- | -- | -- | -- |
| Johnson-Lynn, 2019 (UK) | Plantar fasciitis/ Y/ 6 mths | 28(32.1428571428571); - | 28(32.1428571428571); - | Leucocyte rich PRP, --Rpt: - | Saline, --Rpt: - | -- | -- | -- | -- | -- | -- |
| Kader, 2012 (UK) | Low back pain/ Y/ 3 mths | 63(46.0); - | Pain, Function | Methyl Prednisolone, Marcaine/Bupivacaine --Rpt: - | -- | Education, physiotherapy | -- | Education, Exercise | -- | -- | -- |
| Kaux, 2019 (Belgium) | patella tendinopathy/ Y/ 3 mths | 33(100); - | 33(100); - | PRP (unclear), --Rpt: - | Exercise, | Hyaluronic compound, --Rpt: Weekly (2x) | Exercise, | -- | -- | -- | -- |
| Kazemi, 2010 (Iran) | Lateral epicondylitis/ -/ - mths | 60(18.3); 47(27-64) | Pain, Function | Whole blood, Lignocaine --Rpt: - | Methyl Prednisolone, Lignocaine --Rpt: - |  | -- | -- | -- | -- | -- |
| Kearney, 2013 (UK) | Achilles tendinopathy/ Y/ 3 mths | 20(35); - | Function | PRP (unclear), --Rpt: - | -- | Exercise, | -- | -- | -- | -- | -- |
| Kesikburun 2013 (Turkey) | RC tendinosis/ Y/ 3 mths | 40(32.5); - | Pain, Function | PRP (unclear), --Rpt: - | Physiotherapy, | Saline, --Rpt: - | Physiotherapy, | -- | -- | -- | -- |
| Kongsgaard, 2009 (Denmark) | Patella tendinopathy/ Y/ 3 mths | 37(100); 32.4(8.8) | Pain, Function | Methyl Prednisolone, Lignocaine --Rpt: Monthly (2x) | -- | Exercise, | -- | Exercise, | -- | -- | -- |
| Krogh, 2013 (Denmark) | Lateral epicondylitis/ Y/ 3 mths | 60(48.3); 45.4(8) | Pain, Function | PRP (unclear), --Rpt: - | Exercise, | Triamcinolone, Lignocaine --Rpt: - | Exercise, | Saline, --Rpt: - | Exercise | -- | -- |
| Krogh, 2016 (Denmark) | achilles tendinopathy/ Y/ 6 mths | 24(NA); - | Pain, Function | PRP (unclear), --Rpt: - | Exercise, | Saline, --Rpt: - | Exercise, | -- | -- | -- | -- |
| Kucuksen, 2013 (Turkey) | lateral epicondylitis/ -/ 3 mths | 82(45.1); - | Pain, Function | Triamcinolone, Lignocaine --Rpt: - | -- | Manual therapy, | -- | -- | -- | -- | -- |
| Lebiedziński, 2015 (Poland) | Lateral epicondylitis/ -/ 1.5 mths | 120(NA); - | Function | *ACP, --Rpt: -* | *Bethamethasone, Lignocaine --Rpt: -* | -- | -- | -- | -- | -- | -- |
| Lewis, 2005 (UK) | Lateral epicondylitis/ -/ - mths | 164(NA); - | Pain | Methyl Prednisolone, Lignocaine --Rpt: - | -- | NSAIDs, | -- | Sham/placebo, | -- | -- | -- |
| Lim, 2018 (Korea) | Lateral epicondylitis/ Y/ 3 mths | 120(NA); - | Pain, Function | PRP (unclear), Lignocaine --Rpt: - | physiotherapy, | -- | physiotherapy, | -- | -- | -- | -- |
| Lin, 2019 (Taiwan) | Supraspinatus tendinopathy/ Y/ 6 mths | 31(NA); - | Pain, Function | Dextrose 20%, --Rpt: - | Saline, --Rpt: - | -- | -- | -- | -- | -- | -- |
| Mansiz-Kaplan, 2019 (Turkey) | Plantar fasciitis/ Y/ 6 mths | 60(25); - | 60(25); - | Prolo, Lignocaine --Rpt: Others (2x) | Saline, Lignocaine --Rpt: Others (2x) | -- | -- | -- | -- | -- | -- |
| Mardani-Kivi, 2013 (Iran) | lateral epicondylitis/ -/ 1.5 mths | 100(41); 44.7(8.8) | Pain, Function | Methyl Prednisolone, --Rpt: - | Orthosis, | Procaine, --Rpt: - | Orthosis, | -- | -- | -- | -- |
| Martin, 2019 (Spain) | Lateral and medial epicondylitis/ -/ 3 mths | 80(NA); - | Pain, Function | PRP (unclear), Lignocaine --Rpt: Fortnightly (2x) | Lignocaine, --Rpt: Fortnightly (2x) | -- | -- | -- | -- | -- | -- |
| Martinez-Montiel, 2015 (Mexico) | Lateral epicondylitis/ Y/ - mths | 60(NA); - | Pain, Function | PRP (unclear), --Rpt: - | Methyl Prednisolone, --Rpt: - | -- | -- | -- | -- | -- | -- |
| Merolla, 2017 (Italy) | Lateral epicondylitis/ Y/ 4 mths | 110(NA); - | Pain, Function | PRP (unclear), --Rpt: Fortnightly (2x) | -- | Surgery, |  | -- | -- | -- | -- |
| Montalvan, 2016 (France) | Lateral epicondylitis/ -/ - mths | 50(68); 47(-) | Pain | ACP, Lignocaine --Rpt: Monthly (2x) | Saline, --Rpt: Monthly (2x) |  | -- | -- | -- | -- | -- |
| Mundla, 2017 (India) | Lateral epicondylitis/ -/ - mths | 150(23.3); - | Function | PRP (unclear), --Rpt: - | physiotherapy, | Methyl Prednisolone, --Rpt: - | physiotherapy, | Saline, --Rpt: - | Physiotherapy, | -- | -- |
| Murtezani, 2015 (Kosovo) | Lateral epicondylitis/ -/ 3 mths | 49(57); 51.3(6.4) | Pain, Function | Triamcinolone, Lignocaine --Rpt: Variable (2x) | -- | physiotherapy, | -- | -- | -- | -- | -- |
| Newcomer, 2001 (US) | Lateral epicondylitis/ -/ - mths | 39(48.7); - | Pain | Bethamethasone, Marcaine/Bupivacaine --Rpt: - | physiotherapy, | Marcaine/Bupivacaine, --Rpt: - | physiotherapy, | -- | -- | -- | -- |
| Peerbooms, 2010 (Netherlands) | Lateral epicondylitis/ -/ 6 mths | 100(48); 47(-) | Pain, Function | Leucocyte rich PRP, Marcaine/Bupivacaine --Rpt: - | physiotherapy, | Triamcinolone, Marcaine/Bupivacaine --Rpt: - | physiotherapy, | -- | -- | -- | -- |
| Petrella, 2010 (Canada) | lateral epicondylitis/ Y/ 3 mths | 331(NA); 49(12) | Pain, Function | Hyaluronic compound, --Rpt: Weekly (2x) | Saline, --Rpt: Weekly (2x) |  | -- | -- | -- | -- | -- |
| Price, 1991 (UK) | lateral epicondylitis/ -/ - mths | 88(NA); - | Pain | Hydrocortisone, Lignocaine --Rpt: as needed | Triamcinolone, Lignocaine --Rpt: as needed | Lignocaine, --Rpt: as needed | -- | -- | -- | -- | -- |
| Rabini, 2012 (Italy) | rotator cuff tendinopathy/ Y/ 3 mths | 92(NA); - | Pain, Function | Methyl Prednisolone, Marcaine/Bupivacaine --Rpt: Fortnightly (3x) | -- | Electomodalities, | -- | -- | -- | -- | -- |
| Raeissadat, 2014 (Iran) | Lateral epicondylitis/ -/ 3 mths | 61(23); 45.3(5.9) | Pain, Function | Leucocyte rich PRP, Lignocaine --Rpt: - | Physiotherapy, | Whole blood, Lignocaine --Rpt: - | Physiotherapy, | -- | -- | -- | -- |
| Rahman, 2017 (Bangladesh) | Lateral epicondylitis/ -/ - mths | 80(39); - | Pain | Triamcinolone, Lignocaine --Rpt: - | Exercise, | -- | Electomodalities, Exercise | -- | -- | -- | -- |
| Rha, 2013 (Korea) | Supraspinatus tendon/ Y/ 6 mths | 39(NA); - | Pain, Function | PRP (unclear), Lignocaine --Rpt: Monthly (2x) | Exercise, | Needling, Lignocaine --Rpt: Fortnightly (2x) | Exercise, | -- | -- | -- | -- |
| Runeson, 2002 (Sweden) | Lateral epicondylitis/ -/ 1 mths | 64(NA); - | Pain | -- | Dexamethasone, | -- | Sham/placebo, | -- | -- | -- | -- |
| Ryan, 2014 (Canada) | Plantar fasciopathy/ Y/ 12 mths | 65(NA); - | Pain, Function | Dexamethasone, Lignocaine --Rpt: - | -- | Exercise, | -- | -- | -- | -- | -- |
| Saartok, 1986 (Sweden) | Lateral epicondylitis/ -/ - mths | 21(NA); 45(-) | Pain | Bethamethasone, --Rpt: - | Sham/placebo, | Sham injection, --Rpt: - | NSAIDs, | -- | -- | -- | -- |
| Sabaah, 2020 (Egypt) | Rotator cuff tendinopathy/ Y/ 3 mths | 60(18); 43 (12) | 60(18); 43 (12) | Prolo, Lignocaine --Rpt: Fortnightly (2x) | PRP (unclear), --Rpt: Fortnightly (2x) | Bethamethasone, --Rpt: Fortnightly (2x) | -- | -- | -- | -- | -- |
| Sahoo, 2020 (India) | Plantar Fasciitis/ -/ 3 mths | 73(42.5); 38(11) | 73(42.5); 38(11) | PRP (unclear), --Rpt: - | Exercise, | Methyl Prednisolone, Lignocaine --Rpt: - | Exercise, | -- | -- | -- | -- |
| Scarpone, 2008 (US) | Lateral epicondylitis/ -/ 6 mths | 24(45.8); 45.7(11) | Pain | Prolo, Lignocaine --Rpt: Monthly (3x) | Saline, --Rpt: Monthly (3x) |  | -- | -- | -- | -- | -- |
| Schoffl, 2017 (Germany) | lateral epicondylitis/ -/ 3 mths | 50(56); 52.6(-) | Pain, Function | ACP, mepivacaine --Rpt: Variable (3x) | Saline, --Rpt: - |  | -- | -- | -- | -- | -- |
| Schwitzguebel, 2019 (Switzerland) | SSP tears/ Y/ 6 mths | 84(NA); - | Pain, Function | PRP (unclear), --Rpt: Monthly (2x) | Saline, --Rpt: Monthly (2x) | -- | -- | -- | -- | -- | -- |
| Scott, 2019 (Canada) | patellar tendinopathy/ Y/ 6 mths | 61(NA); - | Pain, Function | Leucocyte rich PRP, --Rpt: - | Exercise, | PRP (unclear), --Rpt: - | Exercise, | Saline, --Rpt: - | Exercise |  |  |
| Singh, 2013 (India) | lateral epicondylitis/ -/ - mths | 60(46.7); - | Function | Whole blood, Lignocaine --Rpt: - | Methyl Prednisolone, Lignocaine --Rpt: - | -- | -- | -- | -- | -- | -- |
| Singh, 2016 (India) | Lateral epicondylitis/ -/ - mths | 65(NA); - | Pain, Function | PRP (unclear), --Rpt: - | Exercise | Methyl Prednisolone, --Rpt: - | Exercise | -- | -- | -- | -- |
| Tetschke, 2015 (Germany) | Lateral epicondylitis/ -/ 3 mths | 56(NA); - | Pain, Function | ACP, --Rpt: Weekly (3x) | physiotherapy | -- | Electomodalities, physiotherapy | -- | -- | -- | -- |
| Thanasas, 2011 (Greece) | lateral epicondylitis/ -/ 3 mths | 28(NA); - | Pain, Function | Whole blood, --Rpt: - | Exercise | Leucocyte rich PRP, --Rpt: - | Exercise | -- | -- | -- | -- |
| Toker, 2008 (Turkey) | Lateral epicondylitis/ -/ - mths | 21(57.1); 45(19-72) | Pain | Methyl Prednisolone, Prilocaine --Rpt: - | NSAIDs | -- | NSAIDs |  |  |  |  |
| Tonks, 2007 (UK) | lateral epicondylitis/ -/ - mths | 48(NA); - | Pain, Function | Triamcinolone, Lignocaine --Rpt: - | -- | Exercise, | Triamcinolone, Lignocaine --Rpt: - | Exercise, | -- | Wait-see policy | -- |
| Tosun, 2015 (Turkey) | Lateral epicondylitis/ -/ 3 mths | 57(NA); - | Pain, Function | Triamcinolone, Prilocaine --Rpt: - | Hyaluronic compound, Prilocaine --Rpt: - |  | -- | -- | -- | -- | -- |
| Uğurlar, 2018 (Turkey) | Chronic plantar fasciitis/ Y/ 12 mths | 158(NA); - | Pain, Function | -- | ESWT, | Prolo, Marcaine/Bupivacaine --Rpt: Weekly (3x) | PRP (unclear), --Rpt: Weekly (3x) | Bethamethasone, Marcaine/Bupivacaine --Rpt: Weekly (3x) | -- | -- | -- |
| Uzunca, 2007 (Turkey) | lateral epicondylitis/ -/ 1.5 mths | 60(NA); - | Pain | Methyl Prednisolone, Prilocaine --Rpt: - | Orthosis, | -- | Electomodalities, Orthosis | -- | Sham/placebo, Orthosis | -- | -- |
| Vetrano, 2013 (Italy) | Patella tendinopathy/ Y/ 6 mths | 46(NA); - | Pain, Function | PRP (unclear), --Rpt: Weekly (2x) | Exercise, | -- | ESWT, Exercise | -- | -- | -- | -- |
| Watts, 2018 (UK) | Lateral epicondylitis/ -/ 6 mths | 83(NA); - | Function | Leucocyte rich PRP, --Rpt: - | -- | Surgery | -- | -- | -- | -- | -- |
| Withrington, 1985 (UK) | Supraspinatus tendonitis/ -/ - mths | 25(NA); - | Pain | Methyl Prednisolone, Lignocaine --Rpt: - | Saline, --Rpt: - | -- | -- | -- | -- | -- | -- |
| Yelland, 2004 (Australia) | Chronic low back pain/ -/ 6 mths | 110(NA); - | Pain, Function | Dextrose 20%, Lignocaine --Rpt: Weekly (6x) | Saline, --Rpt: Fortnightly (6x) | -- | -- | -- | -- | -- | -- |
| Yelland, 2011 (Australia) | Achilles tendinosis/ Y/ 1.5 mths | 43(NA); - | Function | Prolo, Lignocaine --Rpt: - | -- | Exercise | Prolo, --Rpt: Weekly (4-12x) | Exercise | -- | -- | -- |
| Yelland, 2019 (Australia) | Lateral epicondylitis/ -/ 1.5 mths | 120(57); 49(8) | Pain, Function | Prolo, Lignocaine --Rpt: Monthly (4x) | -- | -- | Physiotherapy, | Prolo, Lignocaine --Rpt: Monthly (4x) | Physiotherapy | -- | -- |
| Yi, 2018 (US) | Lateral epicondylitis/ -/ 1.5 mths | 33(39); 48(9) | Pain, Function | Methyl Prednisolone, Lignocaine --Rpt: - | Physiotherapy | -- | Manual therapy, physiotherapy | -- | Orthosis, physiotherapy | -- | -- |

Note:

- =Not specified/ Data unavailable; ACP= autologous conditioned plasma; mths = months; NSAIDs= non-steroidal anti-inflammatories; Prolo= prolothereapy; Rpt= repeat;
