# Supplementary material for: Efficacy of prolotherapy in comparison to other therapies for chronic soft tissue injuries: A systematic review and network meta-analysis
Source: PLoS One. 2021 May 26;16(5):e0252204. doi: 10.1371/journal.pone.0252204 (PMC8153441; doi:10.1371/journal.pone.0252204)
Supplement: S4 Table — (DOCX) [file pone.0252204.s004.docx]

**S4 Table. GRADE assessment of randomised controlled trials performing direct comparisons between prolotherapy and other therapies**

**Question**: Relative efficacy of Prolotherapy in comparison to other treatments for pain at 6 months

| **Certainty assessment** | | | | | | | **S**ummary of findings | | | |
| --- | --- | --- | --- | --- | --- | --- | --- | --- | --- | --- |
| **№ of studies** | **Study design** | **Risk of bias** | **Inconsistency** | **Indirectness** | **Imprecision** | **Other considerations** | **№ of patients** | | **Effect** | **Certainty** |
|  |  |  |  |  |  |  | **Prolotherapy** | **Control** | **Absolute**  (95% CI) |  |
| **vs Placebo at 6 months (assessed with: self-reported pain scale)** | | | | | | | | | | |
| 5 | randomised trials | serious ^a^ | serious ^b,c,d^ | not serious ^e^ | serious ^f^ | none | 183 | 204 | SMD **1.07 SD higher** (0.3 lower to 1.9 higher) | ⨁◯◯◯  VERY LOW |
| **vs Corticosteroid at 6 months (assessed with: self-reported pain scale)** | | | | | | | | | | |
| 4 | randomised trials | serious | not serious | not serious | serious ^f^ | all plausible residual confounding would suggest spurious effect, while no effect was observed ^g^ | 99 | 102 | SMD **0.7 SD higher** (0.4 higher to 1 higher) | ⨁⨁⨁◯  MODERATE |
| **vs blood products at 6 months (assessed with: self-reported pain scale)** | | | | | | | | | | |
| 1 | randomised trials | Very serious ^a^ | not serious | not serious | serious ^f^ | all plausible residual confounding would suggest spurious effect, while no effect was observed ^g^ | 60 | 59 | SMD **0.8 SD higher** (1.2 lower to 2.6 higher) | ⨁⨁⨁◯  MODERATE |
| **vs Non-injectables at 6 months (assessed with: self-reported pain scale)** | | | | | | | | | | |
| 3 | randomised trials | serious ^a^ | serious ^d,h^ | not serious | serious ^f^ | all plausible residual confounding would suggest spurious effect, while no effect was observed ^g^ | 153 | 147 | SMD **0.23 SD lower** (0.4 lower to 0.8 higher) | ⨁⨁◯◯  LOW |

**CI=** Confidence interval; SMD**=** Standardised mean difference

***Explanations***

a. More than half of the included studies were rated as moderate/high risk on Cochrane 2.0 overall bias

b. Significant inconsistency between direct and indirect evidence for numerous comparisons

c. Confidence interval of some direct comparison did not overlap each other as shown in forest plot by study design

d. Placebos/ controls are not homogeneous

e. Placebos are not active treatments and are used as surrogates to standardise comparisons between active treatments

f. Confidence interval of treatment effect either crossed '0" and/or are very wide (Cohen d >2).

g. There are too few studies available for evaluation. However, studies of head-to-head comparison is less likely to suffer from publication bias as placebo controlled studies.

h. The time point measurement ranges widely.
