# Supplementary material for: Efficacy of prolotherapy in comparison to other therapies for chronic soft tissue injuries: A systematic review and network meta-analysis
Source: PLoS One. 2021 May 26;16(5):e0252204. doi: 10.1371/journal.pone.0252204 (PMC8153441; doi:10.1371/journal.pone.0252204)
Supplement: S1 Fig — A = blood product; C = botulinum toxin; D = corticosteroid, F = hyaluronic acid; H = non-injections; I = placebo. (DOCX) [file pone.0252204.s005.docx]

**S1 Fig. Funnel plot for placebo-controlled trials**

A= blood product; C= botulinum toxin; D= corticosteroid, F= hyaluronic acid; H= non-injections; I= placebo
