# Supplementary material for: Efficacy of prolotherapy in comparison to other therapies for chronic soft tissue injuries: A systematic review and network meta-analysis
Source: PLoS One. 2021 May 26;16(5):e0252204. doi: 10.1371/journal.pone.0252204 (PMC8153441; doi:10.1371/journal.pone.0252204)
Supplement: S2 Fig — Note: BP = blood product; BPcombo = blood product combination therapy; Botox = botulinum toxin; CS = corticosteroid; CScombo = corticosteroid combination therapy; HA = hyaluronic acid; Noninj = non-injections; Pcb = placebo; Prolo = prolotherapy. (DOCX) [file pone.0252204.s006.docx]

**S2 Fig. Sensitivity and subgroup analysis by change score, time points, region and study bias**

1. Change Score

1. Time dependent
2. Overall

1. Elbow

1. Shoulder

1. RoB- Randomization

Note: BP= blood product; BPcombo= blood product combination therapy; Botox= botulinum toxin; CS= corticosteroid; CScombo= corticosteroid combination therapy; HA= hyaluronic acid; Noninj= non-injections; Pcb= placebo; Prolo= prolotherapy
