# Supplementary material for: Efficacy of prolotherapy in comparison to other therapies for chronic soft tissue injuries: A systematic review and network meta-analysis
Source: PLoS One. 2021 May 26;16(5):e0252204. doi: 10.1371/journal.pone.0252204 (PMC8153441; doi:10.1371/journal.pone.0252204)
Supplement: S3 Fig — (DOCX) [file pone.0252204.s007.docx]

**S3 Fig. Secondary analysis (alternative treatment grouping)**

 


Note: BP= blood product; Botox= botulinum toxin; -combo= denotes combination therapy with non-injections; combination= injection + noninjection; CS= corticosteroid; HA= hyaluronic acid; Inj= injection; LR-PRP=leucocyte-rich PRP; Noninj= non-injections; Pcb= placebo; Pcb1= injection placebo; Pcb2= noninjection placebo; Prolo= prolotherapy; PRP= platelet-rich-plasma; WB= whole blood
