# Supplementary material for: Efficacy of prolotherapy in comparison to other therapies for chronic soft tissue injuries: A systematic review and network meta-analysis
Source: PLoS One. 2021 May 26;16(5):e0252204. doi: 10.1371/journal.pone.0252204 (PMC8153441; doi:10.1371/journal.pone.0252204)
Supplement: S5 Fig — A. Network sidesplit. *The results are derived from direct evidence only. Note: A = blood product; B = blood product combination therapy; C = botulinum toxin; D = corticosteroid; E = corticosteroid combination therapy; F = hyaluronic acid; G = needling; H = non-injections; I = placebo; J = prolotherapy; K = surgery; L = wait-see policy; Yellow = p<0.05 indicating significant difference between direct and indirect estimates. B. Forest plot by design. Note: BP = blood product; BPcombo = blood product combination therapy; Botox = botulinum toxin; CS = corticosteroid; CScombo = corticosteroid combination therapy; HA = hyaluronic acid; Noninj = non-injections; Pcb = placebo; Prolo = prolotherapy. Yellow: Direct estimate from A-D-I (blood product—corticosteroid—placebo) studies did not overlap with the overall estimates for placebo-blood product comparison. Direct estimates from A-D (blood product—corticosteroid) studies did not overlap with the estimates from A-D-I studies and from overall. C. Inconsistency model for primary analysis. Note: BP = blood product; BPcombo = blood product combination therapy; Botox = botulinum toxin; CS = corticosteroid; CScombo = corticosteroid combination therapy; HA = hyaluronic acid; Noninj = non-injections; Pcb = placebo; Prolo = prolotherapy. (DOCX) [file pone.0252204.s009.docx]

**S5 Figure. Validation: Network sidesplit, Forest plot by design and Inconsistency model of primary analysis**

1. Network sidesplit


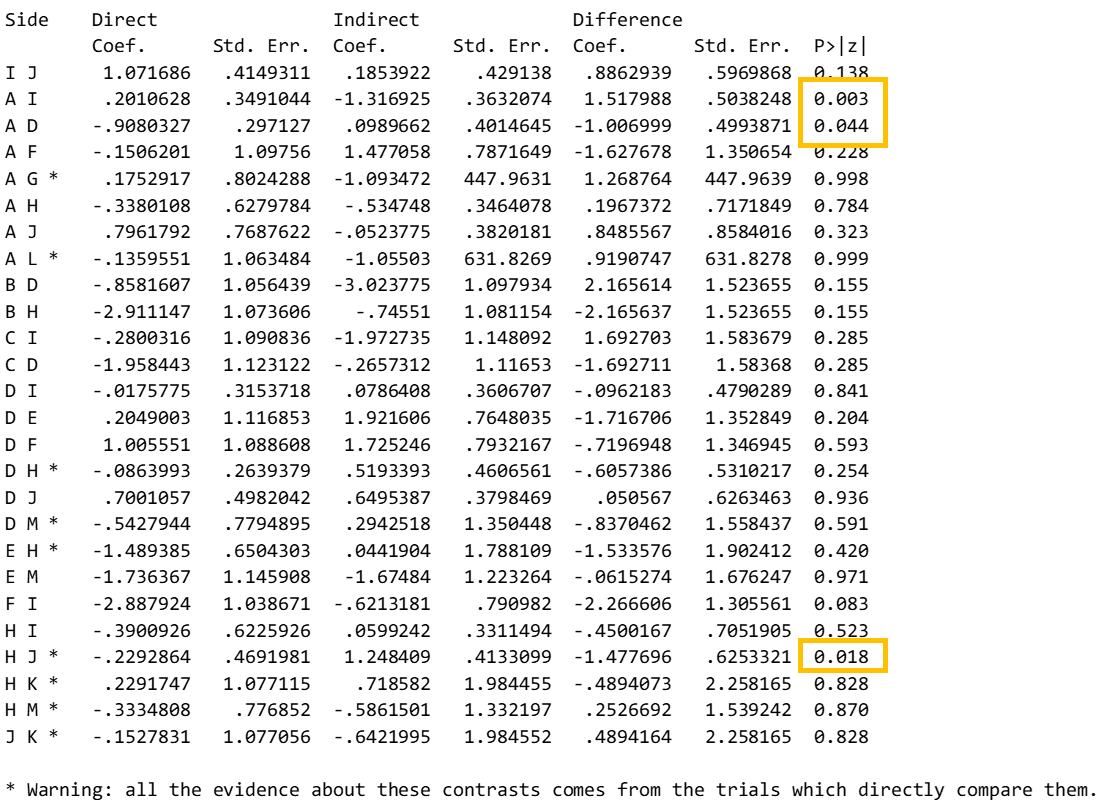


* The results are derived from direct evidence only.

Note: A= blood product; B= blood product combination therapy; C= botulinum toxin; D= corticosteroid; E= corticosteroid combination therapy; F= hyaluronic acid; G= needling; H= non-injections; I= placebo; J= prolotherapy; K= prolocombo; L= surgery; M=wait-see policy; Yellow= p<0.05 indicating significant difference between direct and indirect estimates

1. Forest plot by design


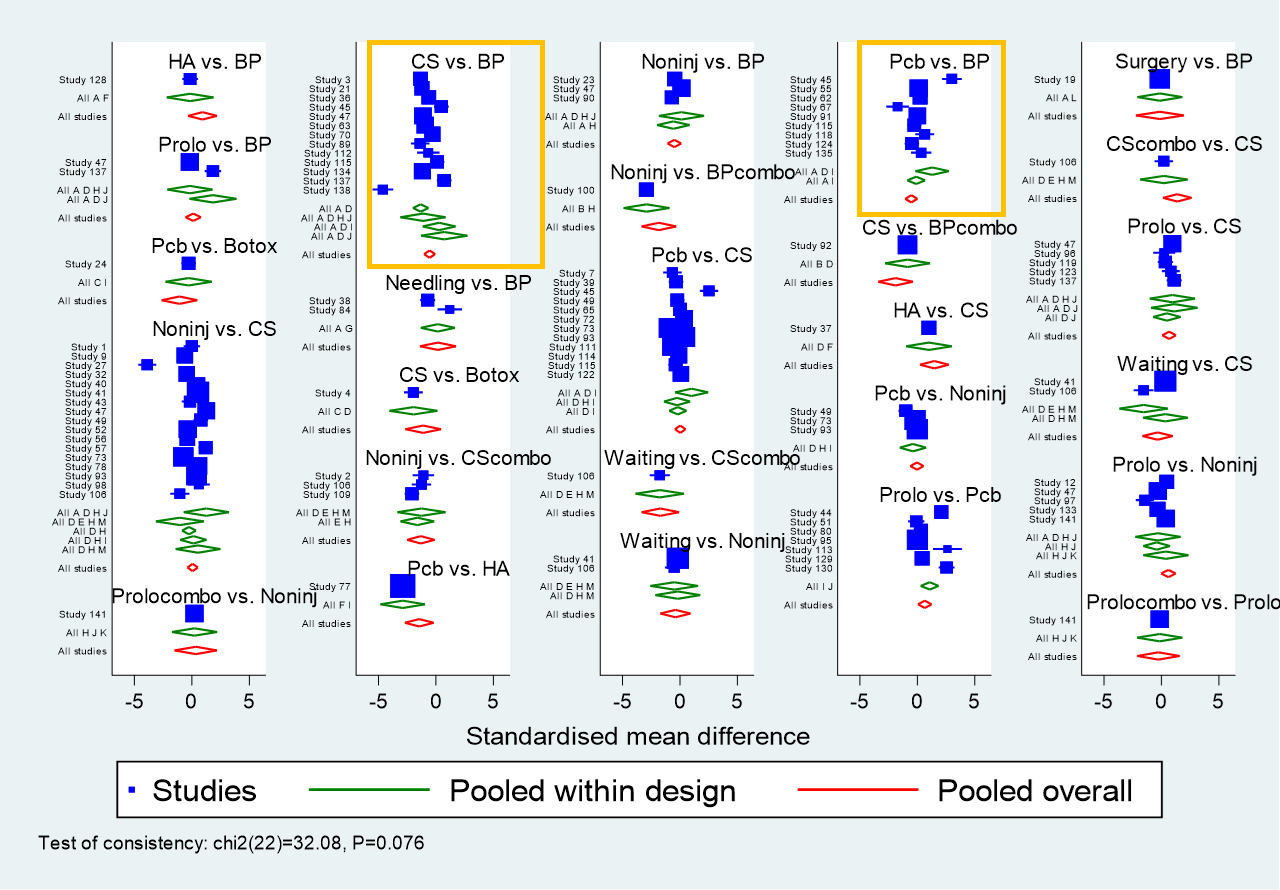


Note: BP= blood product; BPcombo= blood product combination therapy; Botox= botulinum toxin; CS= corticosteroid; CScombo= corticosteroid combination therapy; HA= hyaluronic acid; Noninj= non-injections; Pcb= placebo; Prolo= prolotherapy

Yellow:

Direct estimate from A-D-I (blood product - corticosteroid - placebo) studies did not overlap with the overall estimates for placebo-blood product comparison (yellow outline).

Direct estimates from A-D (blood product - corticosteroid) studies did not overlap with the estimates from A-D-I studies and from overall

1. Inconsistency model for primary analysis

Note: BP= blood product; BPcombo= blood product combination therapy; Botox= botulinum toxin; CS= corticosteroid; CScombo= corticosteroid combination therapy; HA= hyaluronic acid; Noninj= non-injections; Pcb= placebo; Prolo= prolotherapy
